# Supplementary material for: The calcium pump PMCA4b promotes epithelial cell polarization and lumen formation
Source: Commun Biol. 2025 Mar 12;8:421. doi: 10.1038/s42003-025-07814-5 (PMC11904214; doi:10.1038/s42003-025-07814-5)
Supplement: Supplementary file 1 — Supplementary Information [file 42003_2025_7814_MOESM1_ESM.pdf]

Supplementary information for

The calcium pump PMCA4b promotes epithelial cell polarization and lumen formation

Sarolta Tóth[1*], Diána Kaszás[1,2,8], János Sónyák[1], Anna-Mária Tőkés[3], Rita Padányi[3,4], Béla Papp[5,6,7], Réka Nagy[1,8], Kinga Vörös[3,8,9], Tamás Csizmadia[10], Attila Tordai[1] and Ágnes Enyedi[1,11*]

[1] Department of Transfusion Medicine, Semmelweis University, Budapest, Hungary

[2] Department of Physiology, Semmelweis University, Budapest, Hungary

[3] Department of Pathology, Forensic and Insurance Medicine, Semmelweis University, Budapest, Hungary.

[4] Department of Biophysics and Radiation Biology, Semmelweis University, Budapest, Hungary

[5] Institut National de la Santé et de la Recherche Médicale, Inserm UMR 1342, Institut de Recherche Saint-Louis, Hôpital Saint-Louis, Paris, France

[6] Institut de Recherche Saint-Louis, Hôpital Saint-Louis, Université de Paris, Paris, France

[7] CEA, DRF-Institut Francois Jacob, Department of Hemato-Immunology Research, Hôpital Saint-Louis, Paris, France

[8] School of PhD Studies, Semmelweis University, Budapest, Hungary

[9]Institute of Translational Medicine, Semmelweis University, Budapest, Hungary

[10] Department of Anatomy, Cell and Developmental Biology, Eötvös Loránd University, Hungary

[11] ELKH-SE Biophysical Virology Research Group, Eötvös Loránd Research Network, Budapest, Hungary

***Corresponding authors:**

Ágnes Enyedi, email: enyedi.agnes@semmelweis.hu

Sarolta Tóth, email: sarolta.toth7@gmail.com

Supplementary Figures

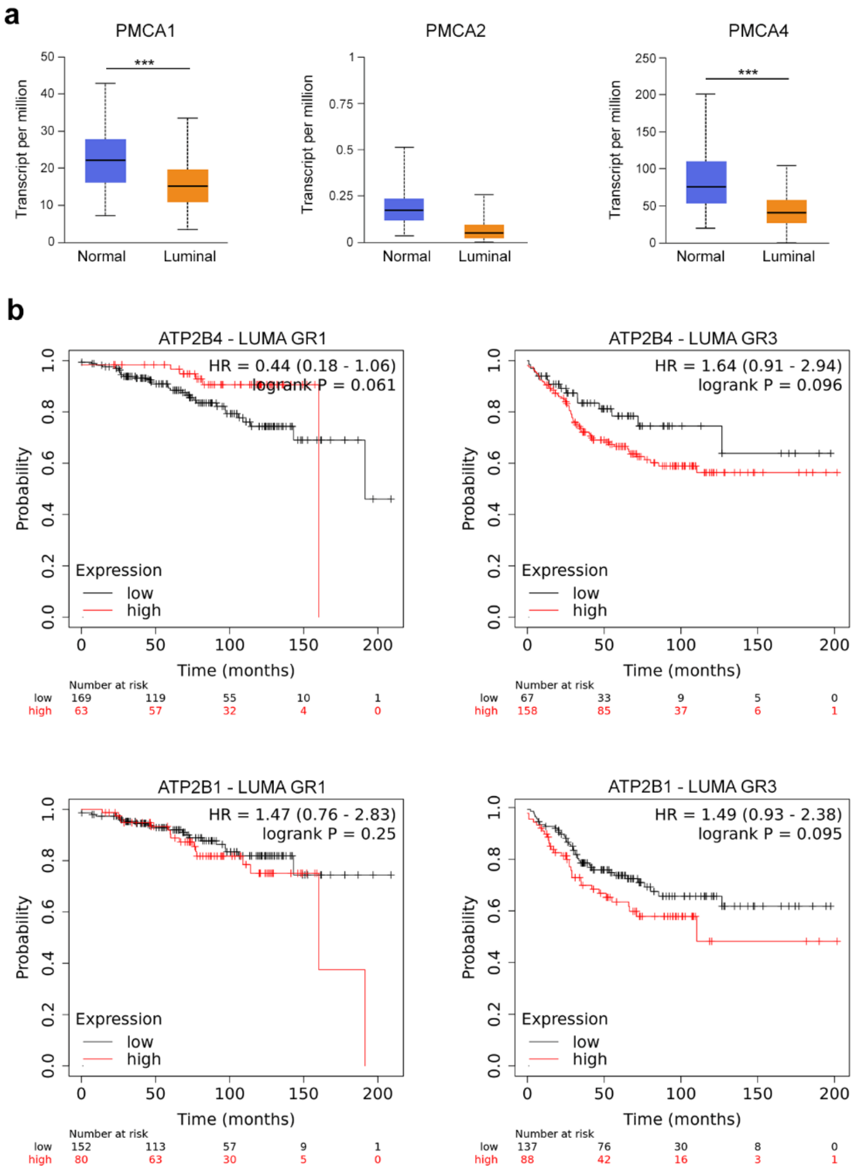

Supplementary Figure 1. PMCA expression in normal and luminal type breast cancer patients. a mRNA level comparison of PMCA1, PMCA2 and PMCA4 in normal breast tissue and luminal breast cancer subtypes. Data derived from The Cancer Genome Atlas, TCGA of the Clinical Proteomic Tumor Analysis Consortium (CPTAC) (http://ualcan.path.uab.edu) database and analyzed by Student's t test; $n_{(normal)}$=114, $n_{(luminal)}$=566; ***$p<0.001$; non-significant differences are not indicated. Error bars show standard deviation. **b** Kaplan–Meier relapse free survival analysis in breast cancer patients with LUMA subtype tumors with low and high *ATP2B4*/PMCA4 and *ATP2B1*/PMCA1 expression levels. GR1 and GR3 indicate grade 2 and 3. Data were collected by the online survival analysis tool (http://www.kmplot.com) using microarray data analysis; p values and the sample sizes are indicated in the figure.

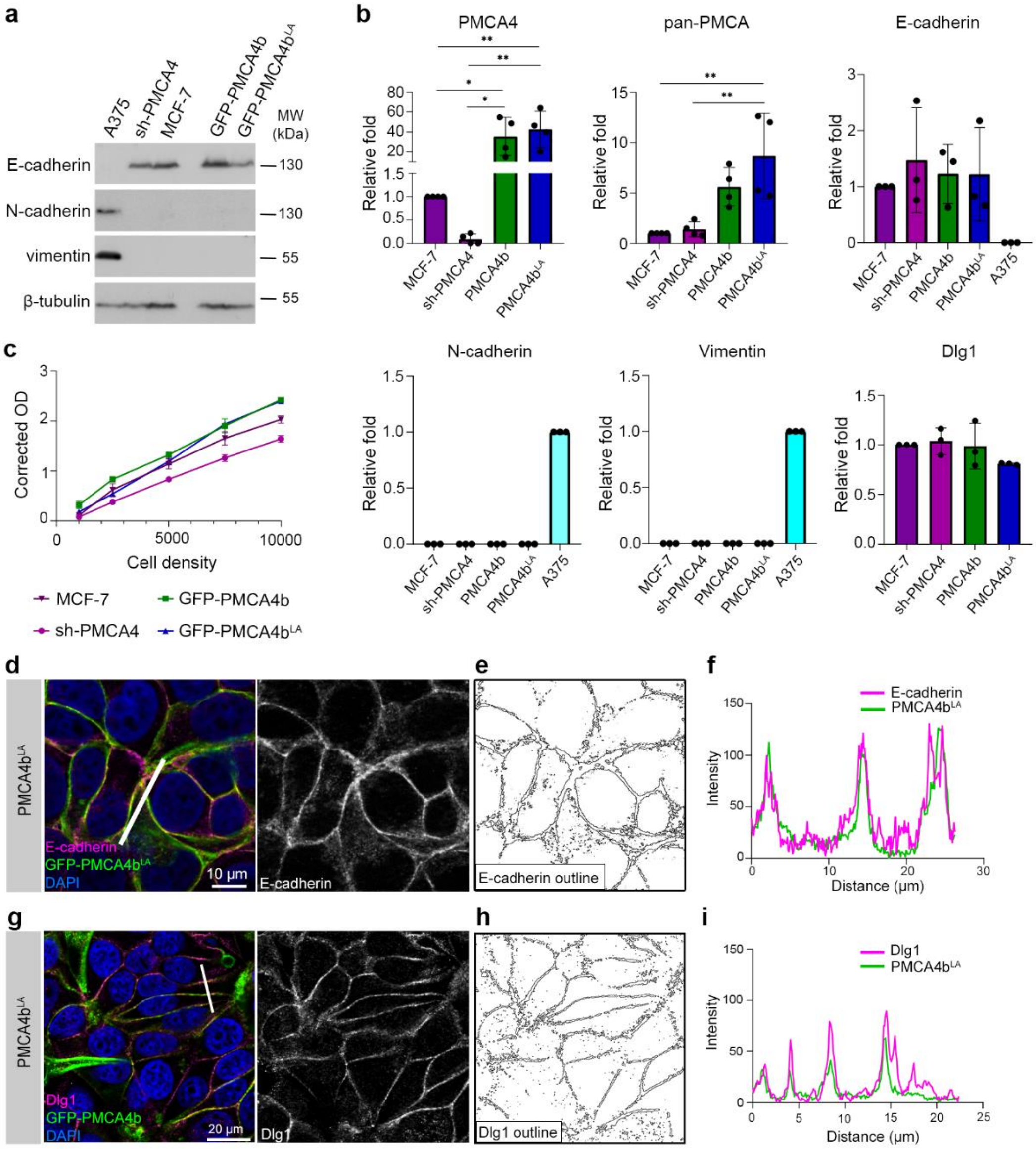

Supplementary Figure 2. **MCF-7 cells display epithelial characteristics. a** Western blot analysis of epithelial (E-cadherin) and mesenchymal marker (N-cadherin, vimentin) proteins in mesenchymal-type A375 cells, and in epithelial-type PMCA4 specific shRNA-expressing, parental, GFP-PMCA4b and GFP-PMCA4b^{LA}-expressing MCF-7 cell lines. A375 cells were used for N-cadherin and vimentin validation. **b** Densitometry analysis of Western blot experiments for PMCA4, pan-PMCA, E-cadherin, N-cadherin, vimentin and Dlg1 proteins in MCF-7 and A375 cells, with the latter serving as a positive control. Graphs display means with standard deviation; data were collected from 3 or 4 independent experiments. Data were analyzed with ordinary one-way ANOVA and Tukey's multiple comparisons tests in the case of PMCA4, pan-PMCA, E-cadherin and Dlg1; adjusted p values: *$p<0.05$; **$p<0.01$; non-significant differences are not labeled. Error bars show standard deviations. The Western blots did show positivity for N-Cadherin and vimentin. **c** Cell density analysis of PMCA4 specific shRNA-expressing, parental, GFP-PMCA4b and MCF-7 cell lines by using sulforhodamine B assay. Error bars show standard deviations. **d** E-cadherin immunostaining of GFP-PMCA4b^{LA}-expressing MCF-7 cells. **e** Outline of the E-cadherin-positive cell compartments generated by the Image J software. **f** Fluorescence intensity profiles of E-cadherin and GFP-PMCA4b across the line shown in D. **g** Dlg1 immunostaining of GFP-PMCA4b^{LA}-expressing MCF-7 cells. **h** Outline of the Dlg1-positive cell compartments generated with the Image J software. **i** Fluorescence intensity profiles of Dlg1 and GFP-PMCA4b^{LA} across the line shown in G.

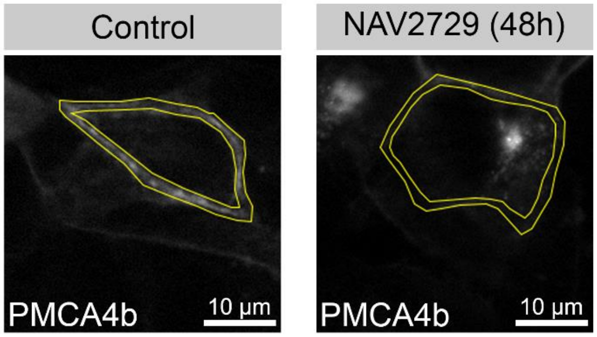

Supplementary Figure 3. Determination of the relative cytosolic GFP-PMCA4b fluorescence signal in control and NAV2729-treated MCF-7 cells. Images show the GFP-PMCA4b fluorescence signal in an individual control and NAV2729-treated PMCA4b-expressing MCF-7 cell. Yellow lines demonstrate areas where intensity was measured for Fig. 6 panel E. The outer line encircles total cell area and the inner line encircles the cytoplasmic area. The proportion of the cytoplasmic PMCA4b signal was calculated by dividing the cytoplasmic signal with the total signal.

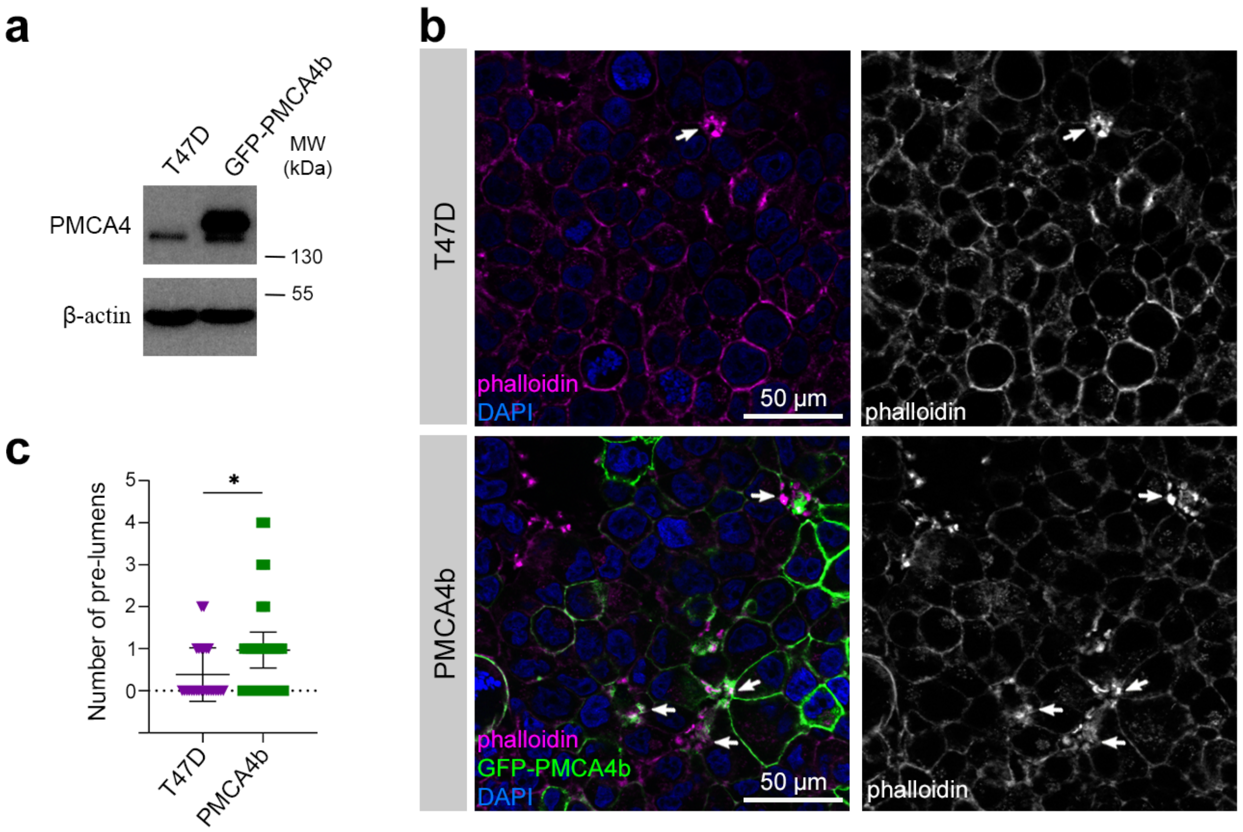

Supplementary Figure 4. PMCA4b overexpression enhances pre-lumen formation in T47D cells. **a** The Western blot shows expression of PMCA4 in parental and GFP-PMCA4b-expressing T47D cells. **b** Phalloidin staining of parental and GFP-PMCA4b-expressing T47D cell lines. White arrows point to pre-lumens. **c** Statistical analysis of pre-lumen formation in parental and GFP-PMCA4b-expressing T47D cells. Graph displays means with 95% coincidence intervals; data were collected from 2 independent experiments, $n_{(T47D)}=26$, $n_{(PMCA4b)}=31$. Data were analyzed with the Mann-Whitney test, p value: $*p<0.05$.

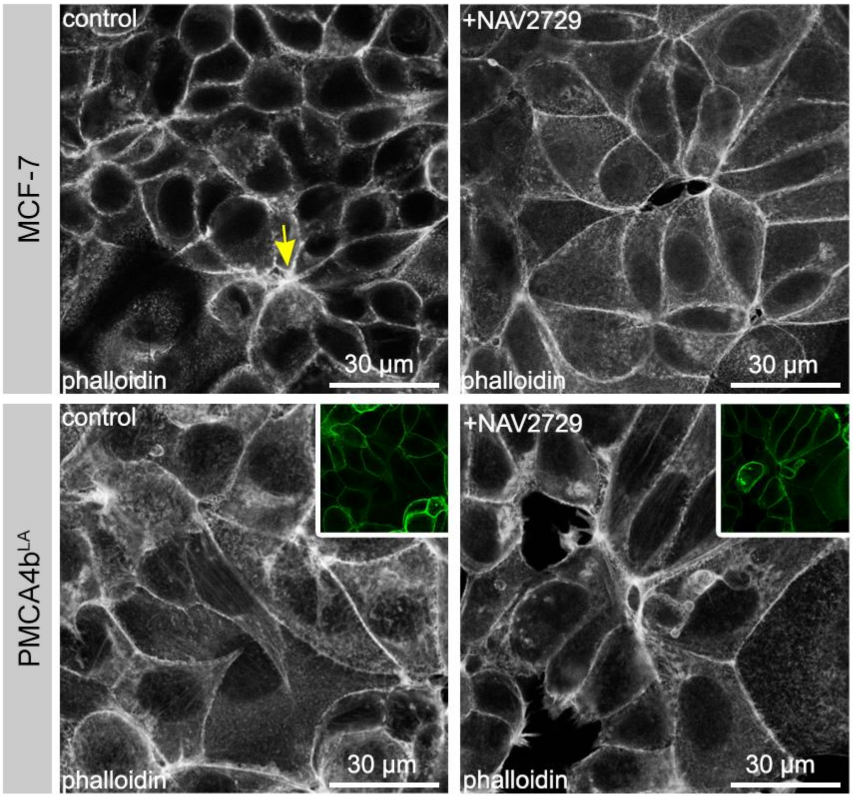

Supplementary Figure 5. Effects of Arf6 inhibition on actin pattern and pre-lumen formation. Phalloidin staining of control and NAV2729-treated parental and GFP-PMCA4b^{LA}-expressing MCF7-cells. Scaled-down insets show GFP-PMCA4b^{LA} signal. Arrow points to a pre-lumen.

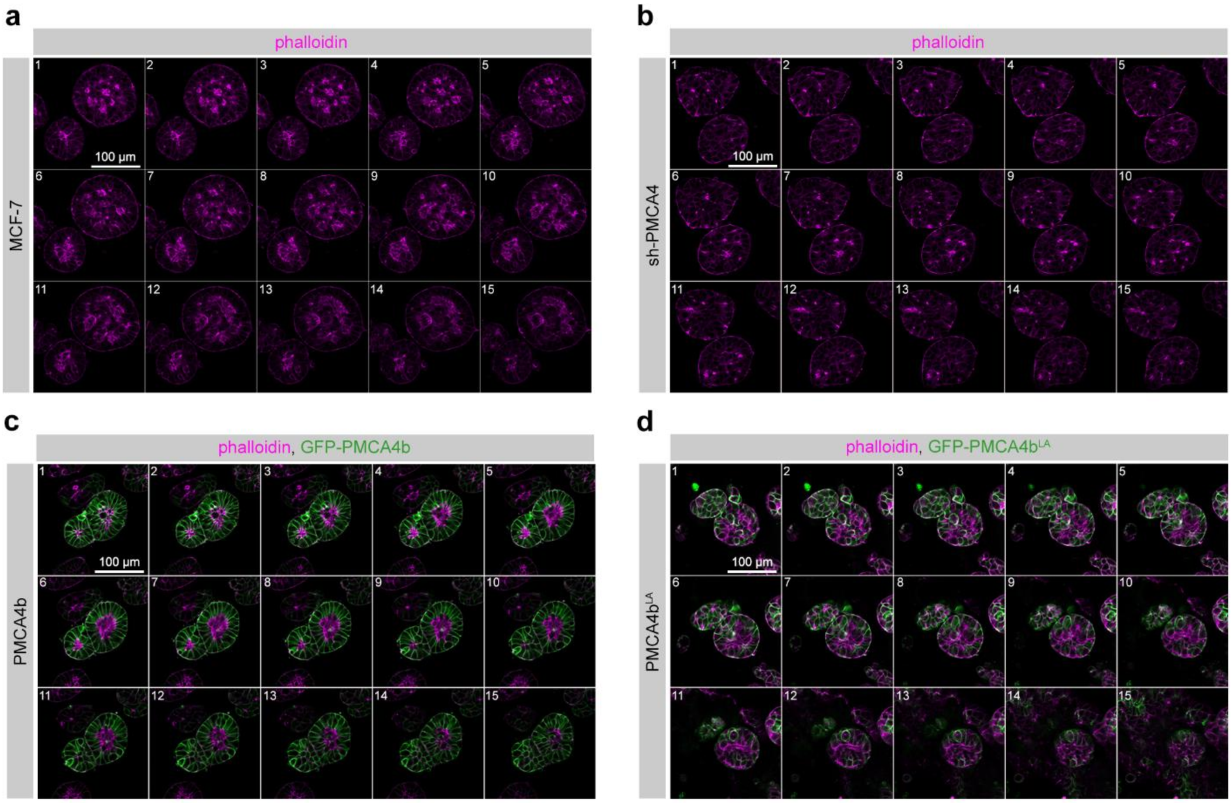

Supplementary Figure 6. **Lumen formation in MCF-7 mammospheres. a-d** Z-stack images of parental, PMCA4-specific shRNA (sh-PMCA4), GFP-PMCA4b and GFP-PMCA4b^{LA}-expressing MCF-7 cells grown in Matrigel for 10 days and stained with phalloidin. The range is 1.25 µm between slices.

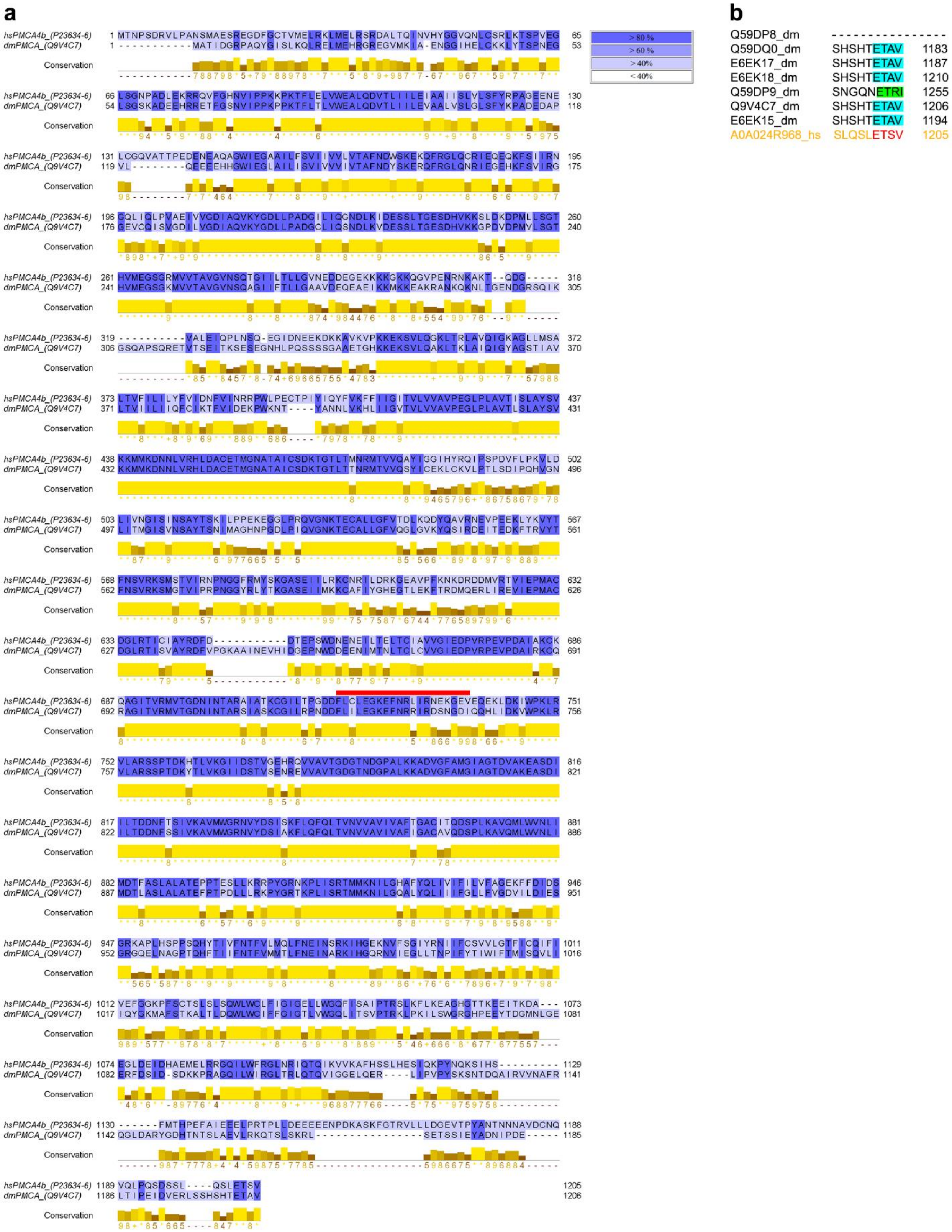

a
b
hsPMCA4b_(P23634-6)
dmPMCA_(Q9V4C7)
Conservation
> 80 %
> 60 %
> 40%
< 40%
Q59DP8_dm ---------------
Q59DQ0_dm SHSHTETAV 1183
E6EK17_dm SHSHTETAV 1187
E6EK18_dm SHSHTETAV 1210
Q59DP9_dm SNGQNETRI 1255
Q9V4C7_dm SHSHTETAV 1206
E6EK15_dm SHSHTETAV 1194
A0A024R968_hs SLQSLETSV 1205

Supplementary Figure 7. Amino acid sequence comparison of human PMCA4b and *Drosophila melanogaster* PMCA sequences. a Alignment of human PMCA4b and *Drosophila melanogaster* (dm)PMCA amino acid sequences using the Jalview software with the region recognized by the 5F10 antibody (located 719-738 in the human amino acid sequence) marked with red line. Purple colors indicate the rate of similarities between amino acids in the same position (ranges are indicated at right) with Percentage Identity Coloring in Jalview. Conservation row in yellow and brown shows conservation rate based on physico-chemical properties. Conserved columns are indicated by asterisks and columns with conserved changes are indicated by "+". **b** Multiple sequence alignment of the C termini of dmPMCA isoforms (black) and human PMCA4b (yellow) by CLUSTAL O (1.2.4). Red color labels the PDZ-binding sequence of human PMCA4b, blue color labels Drosophila sequences with only 1 and green color labels 2 amino acid difference from human sequence.

Supplementary Table

Panel	Genotype
A	w^{1118} (homozygous)
B	w^{1118}; *PBac(681.P.FSVS-1)PMCACPTI001995 (PMCA-GFP)* (homozygous)
C	*da-Gal4* / w^{1118}
	da-Gal4 / $PMCA^{2165R\text{-}3}$
D-G	*fkh-Gal4* / w^{1118}
	fkh-Gal4 / $PMCA^{2165R\text{-}3}$

Genotypes of *Drosophila melanogaster* larvae used in generating data for Figure 7.
