# Supplementary material for: The calcium pump PMCA4b promotes epithelial cell polarization and lumen formation
Source: Commun Biol. 2025 Mar 12;8:421. doi: 10.1038/s42003-025-07814-5 (PMC11904214; doi:10.1038/s42003-025-07814-5)
Supplement: Supplementary file 2 — Description of Additional Supplementary Materials [file 42003_2025_7814_MOESM2_ESM.pdf]

Description of Additional Supplementary Files

File name: Supplementary Data 1

Description: Clinicopathological characteristics of the breast cancer patients' samples. ER (estrogen receptor), PgR (progesterone receptor), HER2 (human erbb2 receptor tyrosine kinase 2) status, and Ki67 (marker of proliferation Ki-67; low means below 5%) index were evaluated by immunohistochemistry. The following data were taken into consideration: age at diagnosis, histological grade, pathologic tumor size (pT), nodal involvement (pN), surrogate breast carcinoma subtypes. T1: the tumor in the breast is 20 millimeters (mm) or smaller in size at its widest area; T2: the tumor is larger than 20 mm but not larger than 50 mm. N0: no cancer was found in the lymph nodes; N1: metastatic lymph nodes. Nonparametric statistical analysis was performed using Kruskal-Wallis and Mann-Whitney tests for comparison of quantitative variables. Statistical significance was defined at $p<0.05$

File name: Supplementary Movie 1

Description: Time-lapse spinning disc confocal microscopy of GFP-PMCA4bexpressing MCF-7 cells after WGA uptake assay. Cells were incubated with WGA for 10 minutes, washed and transferred to a 37 °C incubator for 20 minutes. After that GFPPMCA4b (green) and WGA (red) signals were captured for 25 minutes with spinning disc confocal microscope. The frame rate is 10 FPS.

File name: Supplementary Movie 2

Description: Time-lapse spinning disc confocal microscopy of PMCA4 specific shRNAi-expressing MCF-7 cells after WGA uptake assay. Cells were incubated with WGA for 10 minutes, washed and transferred to a 37 °C incubator for 20 minutes. After that WGA (red) signal were captured for 25 minutes with spinning disc confocal microscope. The frame rate is 10 FPS

File name: Supplementary Movie 3

Description: Time-lapse spinning disc confocal microscopy of GFP-PMCA4bLAexpressing MCF-7 cells after WGA uptake assay. Cells were incubated with WGA for 10 minutes, washed and transferred to a 37 °C incubator for 20 minutes. After that GFPPMCA4bLA (green) and WGA (red) signals were captured for 25 minutes with spinning disc confocal microscope. The frame rate is 10 FPS.
