# Supplementary material for: The calcium pump PMCA4b promotes epithelial cell polarization and lumen formation
Source: Commun Biol. 2025 Mar 12;8:421. doi: 10.1038/s42003-025-07814-5 (PMC11904214; doi:10.1038/s42003-025-07814-5)
Supplement: Supplementary file 9 — Reporting Summary [file 42003_2025_7814_MOESM9_ESM.pdf]

nature portfolio

Corresponding author(s): Sarolta Tóth, Ágnes Enyedi

Last updated by author(s): Feb 6, 2025

Reporting Summary

Nature Portfolio wishes to improve the reproducibility of the work that we publish. This form provides structure for consistency and transparency in reporting. For further information on Nature Portfolio policies, see our Editorial Policies and the Editorial Policy Checklist.

Statistics

For all statistical analyses, confirm that the following items are present in the figure legend, table legend, main text, or Methods section.

n/a	Confirmed	
☐	☒	The exact sample size (*n*) for each experimental group/condition, given as a discrete number and unit of measurement
☐	☒	A statement on whether measurements were taken from distinct samples or whether the same sample was measured repeatedly
☐	☒	The statistical test(s) used AND whether they are one- or two-sided<br>*Only common tests should be described solely by name; describe more complex techniques in the Methods section.*
☐	☒	A description of all covariates tested
☐	☒	A description of any assumptions or corrections, such as tests of normality and adjustment for multiple comparisons
☐	☒	A full description of the statistical parameters including central tendency (e.g. means) or other basic estimates (e.g. regression coefficient) AND variation (e.g. standard deviation) or associated estimates of uncertainty (e.g. confidence intervals)
☐	☒	For null hypothesis testing, the test statistic (e.g. *F*, *t*, *r*) with confidence intervals, effect sizes, degrees of freedom and *P* value noted<br>*Give P values as exact values whenever suitable.*
☒	☐	For Bayesian analysis, information on the choice of priors and Markov chain Monte Carlo settings
☒	☐	For hierarchical and complex designs, identification of the appropriate level for tests and full reporting of outcomes
☐	☒	Estimates of effect sizes (e.g. Cohen's *d*, Pearson's *r*), indicating how they were calculated

Our web collection on statistics for biologists contains articles on many of the points above.

Software and code

Policy information about availability of computer code

Data collection	To compare the prognostic impact of ATP2B4 (PMCA4) and ATP2B1 (PMCA1) expression the publicly available database KM Plotter Online Tool (http://www.kmplot.com) was used. Data for PMCA4 (NP_001675.3:S328) and PMCA1 (NP_001673.2:S1182) protein expression in normal and luminal breast cancer subtypes were derived from the Clinical Proteomic Tumor Analysis Consortium (CPTAC) and analyzed by using the UALCAN web portal (https://ualcan.path.uab.edu/analysis-prot.html). Data for mRNA level comparison of ATP2B1, ATP2B2 and ATP2B4 were derived from The Cancer Genome Atlas, TCGA of the Clinical Proteomic Tumor Analysis Consortium (CPTAC) database. For Homo sapiens PMCA4b and Drosophila melanogaster PMCA amino acid sequence comparison the P23634-6 and Q9V4C7 sequences were used from UniProt (https://www.uniprot.org/). 109 formalin fixed paraffin embedded HR+ and 4 normal breast tissue samples obtained after breast reduction surgery were selected. The cases were diagnosed at the Department of Pathology, Forensic and Insurance Medicine, Semmelweis University, Hungary between 2000 and 2010. Clinicopathological data of the patients were obtained from the files of the Semmelweis University, 2nd Dept. of Pathology and from the Semmelweis University Health Care Database with the permission of the Hungarian Medical Research Council (ETT-TUKEB 14383/2017).
Data analysis	For fluorescent cell evaluation we used ImageJ 1.54f, for statistical analysis we used GraphPad Prism 8.0.1, MiniTab v.21 and SPSS (IBM), for fluorescent imaging we used ZEISS Efficient Navigation 2 (ZEN 2) and iMSPECTOR software (Abberior Instruments GmbH), for electronmicroscope imaging we used iTEM software (Olympus), for picture editing we used Adobe Photoshop 22.4.1., for amino acid sequence alignment we used Jalview 2.11.3.1 software and forPMCA4 IHC scoring we used the slide-viewing software CaseViewer (CaseViewer 2.3.0.99276, 3DHISTECH Ltd., Hungary).

For manuscripts utilizing custom algorithms or software that are central to the research but not yet described in published literature, software must be made available to editors and reviewers. We strongly encourage code deposition in a community repository (e.g. GitHub). See the Nature Portfolio guidelines for submitting code & software for further information.

Data

Policy information about availability of data

All manuscripts must include a data availability statement. This statement should provide the following information, where applicable:

- Accession codes, unique identifiers, or web links for publicly available datasets
- A description of any restrictions on data availability
- For clinical datasets or third party data, please ensure that the statement adheres to our policy

Data are available under the provided links: https://doi.org/10.6084/m9.figshare.26077291, https://doi.org/10.6084/m9.figshare.26068924, https://doi.org/10.6084/m9.figshare.28360466, https://doi.org/10.6084/m9.figshare.28202549. Additional data are available upon request.

Human research participants

Policy information about studies involving human research participants and Sex and Gender in Research.

Reporting on sex and gender	109 luminal breast carcinoma cases obtained from female patients after surgery were included.
Population characteristics	The following data were taken into consideration: age at diagnosis, histological grade, pathologic tumor size, nodal involvement, surrogate breast carcinoma subtypes.
Recruitment	Clinicopathological data of the patients were obtained from the files of the Semmelweis University, 2nd Dept. of Pathology and from the Semmelweis University Health Care Database.
Ethics oversight	Hungarian Medical Research Council (ETT-TUKEB 14383/2017) permission.

Note that full information on the approval of the study protocol must also be provided in the manuscript.

Field-specific reporting

Please select the one below that is the best fit for your research. If you are not sure, read the appropriate sections before making your selection.

☒ Life sciences ☐ Behavioural & social sciences ☐ Ecological, evolutionary & environmental sciences

For a reference copy of the document with all sections, see nature.com/documents/nr-reporting-summary-flat.pdf

Life sciences study design

All studies must disclose on these points even when the disclosure is negative.

Sample size	109 breast carcinoma cases and 4 healthy samples were included. For PMCA1 and PMCA4 protein expression analysis n(PMCA4b/normal)=18, n(PMCA4b/luminal)=64, n(PMCA1b/normal)=18, n(PMCA4b/luminal)=64 samples were available from Clinical Proteomic Tumor Analysis Consortium (CPTAC) (http://ualcan.path.uab.edu). For ATP2B1, ATP2B2 and ATP2B4 mRNA expression analysis n(ATP2B1/normal)=114, n(ATP2B1/luminal)=566, n(ATP2B2/normal)=114, n(ATP2B2/luminal)=566, n(ATP2B4/normal)=114, n(ATP2B4/luminal)=566 were available from the Cancer Genome Atlas, TCGA of the Clinical Proteomic Tumor Analysis Consortium (CPTAC). Kaplan–Meier relapse free survival analysis included n(ATP2B4/LUMA-GR2/low)=97, n(ATP2B4/LUMA-GR2/high)=297, n(ATP2B4/LUMB1/low)=99, n(ATP2B4/LUMB1/high)=301, n(ATP2B4/LUMB2/low)=150, n(ATP2B4/LUMB2/high)=108, n(ATP2B1/LUMA-GR2/low)=257, n(ATP2B1/LUMA GR2/high)=254, n(ATP2B1/LUMB1/low)=239, n(ATP2B1/LUMB1/high)=161, n(ATP2B1/LUMB2/low)=89, n(ATP2B1/LUMB2/high)=169 samples from KMplotter (http://www.kmplot.com). In in vitro cell experiments we analyzed data form 2 independent experiments at least, sample size is indicated in figure legends that was 8 at least, except in the case of densitometry where sample size was 4 or 3. In Drosophila salivary gland experiment we examined 10 control and 10 PMCA silenced salivary glands from different animals.
Data exclusions	Human breast carcinoma cases with no patient follow-up data were excluded from the initial cohort. In vesicle counting non-confluent cell images were excluded, because confluency affects PMCA4 localization. In Drosphila salivary gland cross section examination the photos that were taken of the proximal section of the gland were excluded. No data were excluded in other parts of the study.
Replication	All experiments were performed at least twice to confirm reproducibility.
Randomization	After reviewing the files of patients diagnozed with breast carcinoma between 2000 and 2010, we had randomly selected the HR+ luminal breast carcinoma cases.
Blinding	Breast carcinoma samples were evaluated by two independent investigators before recording the clinical data. The evaluation of fluorescence images were also performed by two independent investigators.

Reporting for specific materials, systems and methods

We require information from authors about some types of materials, experimental systems and methods used in many studies. Here, indicate whether each material, system or method listed is relevant to your study. If you are not sure if a list item applies to your research, read the appropriate section before selecting a response.

Materials & experimental systems

n/a	Involved in the study
☐	☒ Antibodies
☐	☒ Eukaryotic cell lines
☒	☐ Palaeontology and archaeology
☐	☒ Animals and other organisms
☒	☐ Clinical data
☒	☐ Dual use research of concern

Methods

n/a	Involved in the study
☒	☐ ChIP-seq
☒	☐ Flow cytometry
☒	☐ MRI-based neuroimaging

Antibodies

Antibodies used	anti-PMCA4 - Supplier name: Merck, Catalog number: P1494, Clone name: JA9, LOT number: 021M1212; anti-pan-PMCA - Supplier name: Merck, Catalog number: MABN1802, Clone number: 5F10; anti-E-cadherin - Supplier name: Cell Signaling, Catalog number: CST 3195, Clone name: 24E10, LOT number: 10/2013; anti-N-cadherin - Supplier name: Santa Cruz Biotechnology, Catalog number: sc-8424, Clone number: D-4, LOT number: E1806; anti-vimentin - Supplier name: Dako Products, Catalog number: M7020, Clone name: VIM 3B4; anti-Dlg1 - Supplier name: Santa Cruz Biotechnology, Catalog number: sc-25661, Clone name: polyclonal, LOT number: J2010; anti-β-actin - Supplier name: Merck, Catalog number: A1978, Clone name: AC-15, LOT number: 084M4770V; anti-Ezrin - Supplier name: Merck, Catalog number: E1281, Clone name: polyclonal, LOT number: 035K4848; anti-Dlg - Supplier name: Developmental Studies Hybridoma Bank, Catalog number: 4F3, Clone name: 4F3, LOT number: 10R9M0-27; anti-β-tubulin - Supplier name: Abcam, Catalog number: ab6046, Clone name: polyclonal, LOT number: 6R192324-3; anti-CD147 - Supplier name: Sino Biological, Catalog number: 10186-R125, Clone name: #125, LOT number: HA12JA2302; anti-HA - Supplier name: Merck, Catalog number: H3663, Clone name: HA-7; anti-GFP - Supplier name: Thermo Fisher Scientific, Catalog number: A10262, Clone name: polyclonal, LOT number: 2738236; Alexa Fluor 594-conjugated goat anti-rabbit - Supplier name: Thermo Fisher Scientific, Catalog number: A11012, Clone name: polyclonal, LOT number: 2307236; Alexa Fluor 594-conjugated goat anti-mouse - Supplier name: Thermo Fisher Scientific, Catalog number: A11005, Clone name: polyclonal, LOT number: 610868; Alexa Fluor 488-conjugated goat anti-chicken - Supplier name: Thermo Fisher Scientific, Catalog number: A11039, Clone name: polyclonal, LOT number: 1008651; Alexa Fluor 594-conjugated goat anti-mouse - Supplier name: Thermo Fisher Scientific, Catalog number: A11020, Clone name: polyclonal, LOT number: 2318440; Peroxidase AffiniPure™ Donkey Anti-Mouse - Supplier name: Jackson ImmunoResearch, Catalog number: 715-035-151, Clone name: polyclonal; Peroxidase AffiniPure™ Donkey Anti-Rabbit - Supplier name: Jackson ImmunoResearch, Catalog number: 715-035-152, Clone name: polyclonal.
Validation	Anti-pan-PMCA, anti-E-cadherin, anti-N-Cadherin, anti-β-actin, anti-ezrin, anti-β-tubulin, anti-CD147 and anti-HA antibodies are validated according to the supplier website page. Anti-PMCA is succesfully used in following articles: PMID 33802790, PMID 24439526 and PMID 32414111; anti Dlg1 in: PMID 22307621, PMID 16793899 and PMID 22378744. For Drosophila anti-Dlg validation several articles are available on the page of the supplier: https://dshb.biology.uiowa.edu/4F3-anti-discs-large. Anti-vimentin, anti N-cadherin and anti-E-Cadherin are validated in recent manuscript.

Eukaryotic cell lines

Policy information about cell lines and Sex and Gender in Research

Cell line source(s)	MCF7 cell line was purchased from American Type Culture Collection (ATCC), its identifier code is: HTB-22. HEK293H cell line was purchased from ATCC, catalog number is CRL-1573. T47D cell line was obtained from NCI Development Therapeutics Program (DCTD Tumor Repository, National Cancer Institute at Frederick, MD).
Authentication	Cell lines were autenthicated by the supplier. Cells were not used after 20 passages.
Mycoplasma contamination	We routinely stain the nuclei of the cells by DNA stain (DAPI) that is useful approach to detect Mycoplasma contamination in cell culture by fluorescence microscopy. Besides this method we use MycoStrip-Mycoplasma Detection Kit to check Myoplasma infection in our cell cultures. To prevent contamination we supplement the cell culturing media with MycoKill reagent (Avidin Biotechnology).
Commonly misidentified lines (See ICLAC register)	We did not use misidentified cell lines.

Animals and other research organisms

Policy information about studies involving animals; ARRIVE guidelines recommended for reporting animal research, and Sex and Gender in Research

Laboratory animals	We used following Drosophila melanogaster strains in this study: The w1118 strain (FlyBase ID: FBst0003605) and da-Gal4 fly line (exact genotype: w*;P(UAS-da.G)52.2, FlyBase ID: FBst0051669) were obtained from the Bloomington Drosophila Stock Center (BDSC), the PMCA2165R-3 RNAi line (stock ID: 2165R-3) was obtained from the Fly Stocks of National Institute of Genetics (Nig-Fly) and the PMCA-GFP line was obtained from Kyoto Drosophila Stock Center (exact genotype: w1118; PBac(681.P.FSVS-1)PMCACPTI001995, DGRC number: 115256). The fkh-Gal4 fly line was provided by Eric H. Baehrecke (University of Massachusetts Medical School, Worcester, MA).
Wild animals	The study does not involved wild animals.
Reporting on sex	The sex of the animals was not of interest in our study.
Field-collected samples	Flies were raised at 25°C on standard cornmeal, yeast and agar containing medium.
Ethics oversight	No ethical approval or guidance was required because Drosophila melanogaster is not subject to ethical concerns.

Note that full information on the approval of the study protocol must also be provided in the manuscript.
